# Do disabilities impede the use of information and communication technologies? Findings of a repeated cross-sectional study – 2003-2015

**DOI:** 10.1186/s13584-018-0260-x

**Published:** 2018-10-26

**Authors:** Sabina Lissitsa, Galit Madar

**Affiliations:** 1 School of Communication, Ariel University, Ariel, Israel; 2 Faculty of Health Sciences, Ariel University, Ariel, Israel

**Keywords:** People with disabilities, Digital divide, Internet adoption, Digital uses

## Abstract

**Background:**

The current research focuses on trends of Internet adoption and digital uses among people with disabilities over a thirteen-year period.

**Methods:**

The study is based on data elicited from a repeated cross-sectional study collected by means of Annual Social Surveys conducted by Israel’s Central Bureau of Statistics between 2003 and 2015. The sample included 95,145 respondents, among them 22,290 respondents with disabilities.

**Results:**

The rate of Internet access and digital uses increased continuously among disabled people; however the gap between them and the population without disabilities was preserved. We found that Internet use depends on a number of socio-economic characteristic. Socio-demographic variables were much more powerful in predicting Internet use vs non-use among the total population, compared to predicting digital uses among Internet users.

**Conclusions:**

Our findings make it possible to identify disadvantaged groups in which disability intersects with low rates of Internet adoption and belonging to unprivileged groups: Arabs, the religious, the elderly, lower SES individuals. The effects of most of these variables did not change in the period under study. Generally, we recommend finding a way to promote courses that focus on promoting digital literacy in general and eHealth literacy in particular in small groups of people of similar age, digital skill level and motor / health problems. Considering the high representation of Arabs among people with disabilities and lower rates of Internet adoption and use among Arabs, it is recommended that efforts continue to increase the scope and quality of Arabic language content published on Israeli eHealth sites. In order to diminish income-based digital divide we recommend providing publicly accessible free information technologies, for example, in community clubs, senior citizen clubs, and independent- and assisted- living projects for the disabled.

## Background

Emerging information and communication technologies (ICT), foremost among them the Internet, may have an especially profound impact on health knowledge, services, and care opportunities available to people with disabilities (hereafter PWD) [9, 69]. For PWD the possibility of nearly instantaneous communication across the globe means being able to tap into sources of health information [22], participate in forums discussing health issues, create new social relations unlike anything available in their physical non-ICT world [12, 14, 39, 63], empower their sense of independence and self-determination[15], as well as improve health outcomes and lower health care costs ([46], Manganello, 2017).

Access to technology and its benefits is not equally distributed between or within nations [36]. *Digital divide* separates those who have access to information and communication technologies and the ability utilize them, and those who do not [13]. In all Western countries, belonging to a vulnerable group (i.e., one with lower education, disabilities, and social isolation) is a strong predictor of non-online access [45]. Within these digitally excluded groups, it has been suggested that PWD are overly represented on the wrong side of the digital divide [83]. Several studies have found that Internet adoption and use among PWD was lower, compared to people without disabilities [6, 25]. Moreover, Internet usage was less probable in the case of people with severe disabilities when compared to people with mild disabilities [25, 30]. Furthermore, in predicting digital use disabilities may intersect with additional risk factors characterized, among other things, by lower rates of Internet penetration [82]. Among those manifesting such factors are economically and socially disadvantaged populations such as ethnic minorities, the elderly and those of low socio-economic status.

According to the World Health Organization (WHO) the percentage of the population aged 15 years or over who experienced some form of disability grew from 15.3% in 2004 to 19.4% in 2010. The disabled population is a major sector of diversity and inequality in society, but analysis of it as such has lagged behind that of other social factors when it comes to digital uses (for exceptions, see [23, 35, 47, 81]). Incorporating this variable into investigations of digital inequality is important because, unlike many other social statuses, disability is one that may become relevant to almost anyone at some point in the life cycle [75].

As far as we know, no research using a large population with disabilities has been conducted that investigates *trends in Internet adoption and use and the effect of socio-demographic variables* on Internet use in the early years of the twenty-first century – a highly important period of mass Internet adoption. This is the purview of the current research.

The main purpose of the current study is to follow the trends in ICT adoption among the population of Israelis with disabilities (compared to the population without disabilities) from 2003 to 2015 and to identify variations in the socio-demographic characteristics that may predict Internet access and digital uses over time.

It is fitting that this type of research is being conducted in Israel, given the nation’s position as a global leader in the adoption of mobile phones, Internet use, and electronic health records [68] on the one hand and the distressful economic situation of its disabled on the other. Most patient care in Israel is provided by means of several advanced, integrated private not-for-profit delivery systems [70] that strive to increase the quality and efficiency of health care using Health Information Technology as a main driver. In this context it may be assumed that those who do not use Internet and acquire the appropriate digital skills required for it may pay a significant price in terms of their health.

With total population of about 9 million people, Israel has 1.6 million PWD, of them 535,500 with high levels of disability [7]. 50,000 of them are wheelchair-bound, some 24,000 are legally blind and 15,000 are deaf. The percentage of PWD is higher among vulnerable population groups: senior citizens (58% of those aged 65+ are PWD), women, Arabs, unemployed, people with low SES [7]. The disability allowance, which was 2239 shekels ($640) in 2000, has risen only slightly, to 2342 shekels ($670), in the past 17 years. A study by the National Insurance Institute indicated that within a decade the poverty rate among the disabled in Israel increased fourfold compared to the general population [85]. After a year of negotiations and dozens of wildcat protests that included blocking roads and snarling traffic, the Knesset in February 2018 raised the monthly stipend to NIS 3700 ($1050). The name of one of the organizations of disabled which initiates road-blocking actions, “Disabled, not half a person”, reflects its approach to the basic right to live with dignity.

## Literature review

### Disability and internet use

The World Health Organization (WHO) includes both medical and societal factors in its definition of disability: ‘[Disability is] a complex phenomenon, reflecting the interaction between features of a person’s body and features of the society in which he or she lives’ (WHO 2017). In contrast to the outdated medical definition of disability, which focused only on individual aspects of impairment, activity limitations and restrictions on participation, this contemporary definition also underscores the *social* aspect. As disability is universal and PWD are part of all societies, contemporary discourse emphasizes the *social model of disability*.

The basic idea of the social model is that PWD are not disabled by their impairments but by the disabling barriers they faced in society [37, 66]. PWD are excluded in many domains of life, with consequences impacting their health and wealth [44, 74]. Viewed from the perspective of the social model, which stresses the interaction between the social environment and individuals with disabilities, it is inevitable that the Internet functions within the boundaries of extant social conditions and inequities [37]. This social understanding of the web highlights the importance of web accessibility for digital participation by individuals with disabilities on the basis of explicit, emancipatory values [52]. However, studies show that PWD do not utilize digital era opportunities. Rather than creating broader inclusion, digital technologies may have the opposite effect and in many cases further isolate people with a range of impairments [34, 43, 64]. PWD are excluded in their own homes from accessing technology due to lack of funds, lack of support or lack of skills to access resources that differ from those used by the non-disabled population [2, 50, 65]. An Israeli survey found that even if chronically ill PWD use Internet, they derive less benefit from it due to their lower digital literacy, compared to people who are younger and without disabilities. In other words, those who need more health information and services are less able to obtain them [62]. Unfortunately, the creators and vendors of new online hardware and software tend not to take PWD into consideration when planning their designs [27, 50]. Moreover, as a consequence of the fast-paced development and evolution of digital media, continuous updating requirements, high financial cost, non-accessible design and poor training from providers, digital and assistive technologies may actually create a new level of social inequality rather than benefiting disabled people, and thus reinforce the digital divide [4, 43, 47, 58].

However from the perspective of the *compensation model* [16] the relationship between disability and the Internet can be viewed as an opportunity. This model postulates that disabled persons are isolated and have low levels of social interaction, creating social interaction needs for which online communication can compensate [10]. In this way digital technologies have the potential to enable PWD to overcome ‘limitations’ in their body in order to improve their life-chances. People who are socially inactive or dissatisfied with their social interactions in the physical non-ITC world tend to use the Internet more frequently, and hence benefit from it more [16]. Scholars reported that while PWD (43%) were less likely to access the Internet when compared with the individuals without disabilities (57%), they spent double the amount of time online. PWD reported that use of the Internet enabled them to better connect with others and obtain information not previously accessible. Thus, 48% reported that Internet improved their quality of life, compared to 27% of individuals without disabilities [78].

Internet access may also increase the sense of independence and self-determination of PWD [15], facilitate lifelong learning and serve as a tool that enables or supports professional activities [28]. From a practical point of view, everyday life of PWD may be substantially improved through access to such online services as e-banking, Internet shopping or simply communicating via e-mail or videoconferencing with families and friends [4, 17, 77]. The benefits of using Internet for health purposes may include new areas of physician–patient interaction and self-care [38, 84]. For those who suffer from limited mobility, use of Internet is sometimes the only way to perform activities which otherwise would be unavailable to them. People with hearing and visual impairments can benefit from available tools that help to circumvent their sensory deficiencies.

### Digital divide

Today, it is customary to separate the digital divide into two levels of inequality: the first distinguishes between those who are connected and those who are not. The second pertains to the surfing patterns used by those connected to Internet, including measurements of different types of Internet uses [20, 40, 71]. Some Internet usage activities are more beneficial or advantageous for users – offering them greater opportunities and resources for advancing their careers, work, education and social status (capital-enhancing uses) than others intended for momentary consumption or entertainment (recreational uses) [21, 41, 60, 80, 87].

In the current study we investigate the first type of digital uses, which includes *human capital-enhancing* forms of Internet use and *social capital-enhancing* forms of Internet use [53–55] which may be especially relevant for people with disabilities. Human capital-enhancing uses refer to Internet surfing for beneficial purposes, including seeking health information, researching products, current events, etc. [42]. Recent studies estimate that 70–80% of Internet users have searched for health-related information online [31, 59]. Furthermore, 75% of Internet users with a chronic condition say their last online health search affected a decision about how to treat an illness or condition [31]. However, seeking information for health purposes raises some causes for concern. The health information provided on many websites is often of low quality, inaccurate and in most cases incomplete [76].

Social capital-enhancing digital uses refer to the ability to communicate with other people by e-mail and social media. The social media create a platform for communications among a dynamic consortium of people utilizing social network sites, forums, discussion groups and blogs in a manner that enables individuals with a common interest to interact continually and to promote different types of benefits [11, 48]. About 90% of Internet users over the age of 50 reported frequent use of social networks such as Facebook and Twitter to find and share a source related to their health [79]. A growing body of literature reports changing modes of physician/patient communication in the digital age: the use of e-mail as an adjunct or alternative to face-to-face patient/doctor visits or more traditional clinic telephone lines [38, 84].

Digital divides tend to mirror preexisting patterns of economic inequality [20, 49, 67]. Differences in access to and forms of Internet use reflect more limited income potential among populations without access to online information or surfing skills, a lack of equality in employment opportunities and more limited social mobility [19, 53, 55, 57]. As a result, the vulnerable social groups, such as ethnic and national minorities, people from low SES, elderly and periphery residents find themselves doubly disadvantaged. In Israel, religiosity can be added to the factors predicting Internet anxiety and lower usage rates [5, 18]. In the Jewish world, most ultra-Orthodox groups tend to be deeply suspicious of all aspects of modern communication technologies that may enable access to undesirable content which may negatively and irreparably damage unique community lifestyles ([61, 88]).

The problems that PWD must deal with in economic, cultural and social aspects of their everyday life and the advantages that the use of computers and the Internet provides transcend national borders. It is crucial for policy makers to understand the overlap of disability dynamics with other limiting socio-economic factors in their efforts to overcome the disadvantages and vulnerability of such groups and to address their specific and unique needs. Accordingly the following research questions may be formulated:

## Research questions

1. What trends in Internet access and digital uses emerged from 2003 to 2015 among Israeli PWD, compared to people without disabilities?
2. Which variables may predict Internet access and digital uses among Israeli PWD, compared to people without disabilities?
3. What changes, if any, were observed over time in patterns of predictability of the effects of socio-demographic variables on Internet access and digital uses among Israelis in both groups?

## Methods

### Source of data

The current research is based on a repeated cross-sectional study. We used data which were collected by means of Annual Social Surveys conducted by Israel’s Central Bureau of Statistics (CBS) in the period between 2003 and 2015. The CBS conducts a social survey annually using different respondents each year. The surveys provide up-to-date information about living conditions and the welfare of the population in Israel. The formulation of all the questions used in the study was identical throughout this period.

CBS interviewers carried out face to face interviews in the field between January and December of each year. The duration of each of the interviews, which were conducted in Hebrew, Russian and Arabic, was about 1 h.

### Population and sampling method

The survey pool population comprises the permanent non-institutional population of Israel aged 20 and older, as well as residents of non-custodial institutions (such as student dormitories, immigrant absorption centers and independent living projects for the elderly). New immigrants are included in the survey population if they have been resident in Israel for at least 6 months.

Each year the CBS sample size was about 7500 persons aged 20 and older, representing about 4.5 million people in that age bracket. The response rate was around 80%. The sample design involved defining groups based on a combination of three demographic variables: population groups (Israeli-born Jews, immigrants and Arabs), age and gender. The expected size of each design group was to be proportional to its size in the population. The social survey samples are based on random selection and the sampling method enables generalization of the results to the entire Israeli population.

We created our database using 13 years of CBS Social Survey data. It included 95,145 respondents, among them 22,290 respondents with physical and health problems which interfere or interfere *greatly* with daily functioning (the PWD group). The socio-demographic characteristics of the sample are presented in Appendix 1.

### Variables

#### Independent variables

*Physical or health problems interfering with day-to-day functioning* were originally measured on scale of 1–5, 1 – do not have physical or health problem, 2 – the problem doesn’t interfere at all, 3 – the problem doesn’t interfere very much, 4 – the problem interferes, 5- the problem interferes greatly. In order to define the PWD group this variable was divided into two groups (categories 4 and 5 are defined as PWD, categories 1, 2 and 3 are defined as people without disabilities).

*Ethnicity* was measured by two dichotomous variables: Arabs and immigrants (those who immigrated to Israel after 1989). Veteran Jews (born in Israel or immigrated before 1989) were the comparison group.

*Gender* was coded 1 for men and 0 for women.

*Age* was measured in five-year categories. This variable was transformed into a continuous variable using the midpoint of each group.

*Religiosity* was measured on a scale of 1–4: 1–Not religious, secular; 2 – Traditional; 3 – Religious, 4 – Very religious.

*Marital status* was measured as a dichotomous variable: 1 – married; 0 – other marital status.

*Number of children* was measured as a continuous variable.

*Area of residence* was coded 1 for center residents (Jerusalem, Tel-Aviv, and Central region) and 0 for periphery residents (North, Haifa, South, Judea, and Samaria).

*Education* was measured by the highest diploma received by the respondent (see Appendix 1).

*Employment* was measured as a dichotomous variable: 1 – works, 0 – doesn’t work.

*Income level* was measured by the item: Last month, what was the total gross income of all members of the household, from all sources: work, pensions, support payments, rents, etc. on a scale of: 1. NIS 2500 (New Israeli Shekels) or less; 2. NIS 2501–4000; 3. NIS 4001–5000; 4. NIS 5001–6500; 5. NIS 6501–8000; 6. NIS 8001–10,000; 7. NIS 10,001–13,000; 8. NIS 13,001–17,000; 9. NIS 17,001–24,000. 10. NIS More than 24,000. This variable was transformed into a continuous variable using the midpoint of each group and divided by 1000. The top income category was recorded as NIS 30,000.

##### Hebrew language proficiency

The respondents were asked: What is the level of your knowledge of the Hebrew language, in speech, reading and writing? The scale was 1–very good, 2–good, 3–moderate, 4–weak, 5–don’t know at all. A combined index for reading, writing and speaking skills in Hebrew was measured on a scale of 1–5 (1–Not at all, 5–Very well) which was constructed as the average of these three language skills. Cronbach Alpha for Proficiency in Hebrew language was .95.

#### Dependent variables

*Using Internet in the last 3 months* was measured by the following item: During the last 3 months, have you made use of the Internet, including e-mail? Internet access was coded as 1 for those who used and 0 for non-users.

*Human capital-enhancing forms of Internet use* were measured by the following item: Did you use a computer during the last 3 months for seeking information?

*Social capital-enhancing forms of Internet use* were measured by the following two items: Did you use a computer during the last 3 months (a) for e-mail? (b) for discussion groups and communications, e.g., chat rooms, forums, Messenger, Skype? (Facebook has been included in the question about social media use since 2010; Twitter since 2012). In each item, users were coded as 1 and non-users as 0. Descriptive results for different forms of Internet use are presented in Appendix 2.

#### Building measures for social capital-enhancing forms of internet use

As the original variables (e-mail usage and social media usage) were measured on a dichotomous scale (0–does not use, 1–uses), the scale for measuring social capital-enhancing forms of Internet use ranges from 0 to 2 (0–does not employ any of the uses; 1–employs only one type of social uses; 2–employs both types of social uses).

#### Control variables

*Wave of data collection* was coded on a scale of 0–12, where 2003 = 0, 2015 = 12.

## Results

We will first present the findings regarding Internet access and use over time and then apply multivariate analysis in order to predict Internet access and digital uses. In predicting digital uses, we focused only on the group of Internet users, excluding all non-users.

### Descriptive findings

#### Internet access over time

**Fig. 1:**
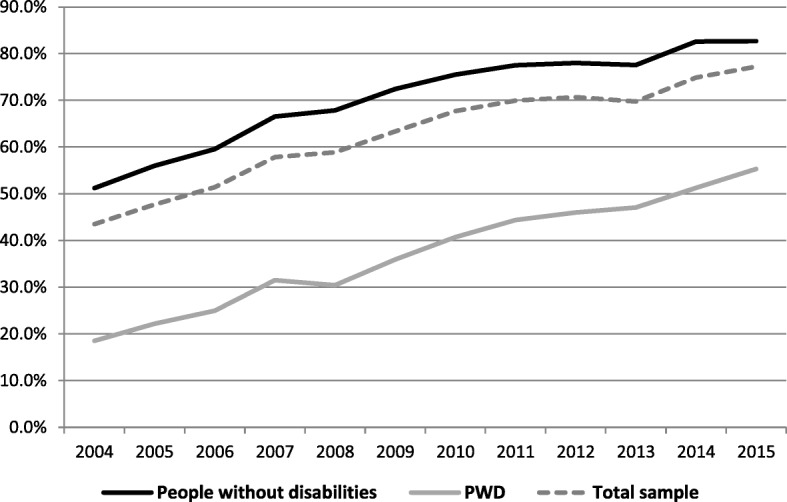
Using the internet in the last 3 months between 2003 and 2015

Figure 1 shows the percentage of Internet access for PWD and people without disabilities and for the total sample.

Figure 1 shows that Internet access among PWD rose from 18% in 2003 to 55% in 2015 (an increase of 37 percentage points). At the same time, the percentage of Internet adopters among the population without disabilities rose from 45 to 83% (an increase of 38 percentage points). Thus, the absolute gap between both groups remained unchanged. The odds ratio was similar between both groups.

**Fig. 2:**
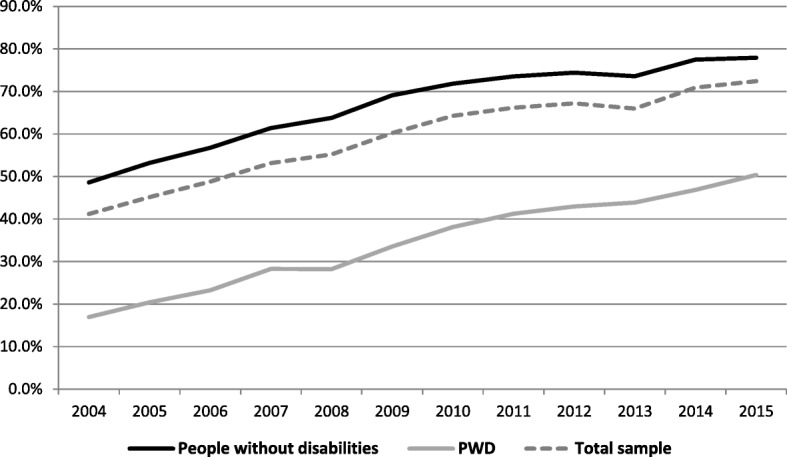
Using the internet for seeking information in the last 3 months between 2003 and 2015

Figure 2 presents the rates of *Internet use for seeking information* in the total sample. The percentage of Internet uses among Internet users is presented in Appendix 2.

Figure 2 shows that information seeking among PWD rose from 15% in 2003 to 50% in 2015 (an increase of 35 percentage points). This digital use among people without disabilities rose from 41 to 78% (an increase of 37 percentage points). However, the odds ratio was slightly more pronounced for PWD (5.6) than for people without disabilities (5.1) as a result of the very low starting point among the former. Comparing Figs. 1 and 2 we can see that the percentage of Internet use for information seeking is only slightly lower than the access rate.

**Fig. 3:**
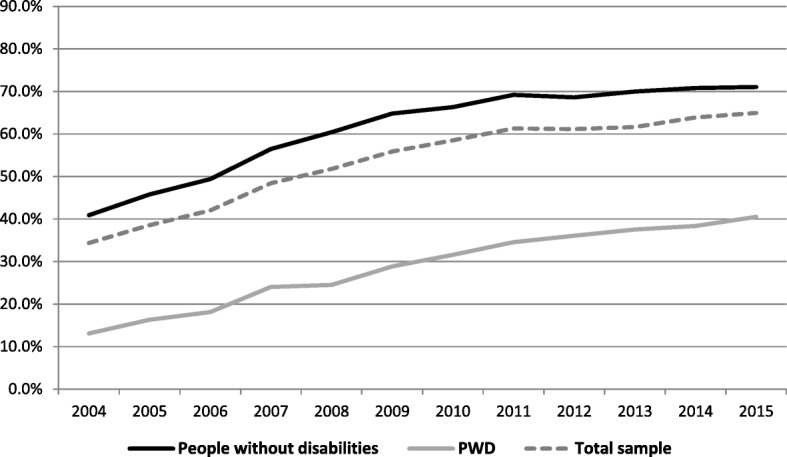
Using the internet for e-mail in the last 3 months between 2003 and 2015

Figure 3 shows that e-mail usage among PWD rose from 12% in 2003 to 41% in 2015 (an increase of 29 percentage points) while the access rate among respondents without disabilities rose from 35 to 71% (an increase of 36 percentage points). However, the odds ratio was slightly more pronounced for the PWD group (4.9) than for the group without disabilities (4.5) as a result of the very low starting point among the former. Comparing Figs. 1 and 3 we can see that the percentage of Internet use for e-mail among PWD is lower than the access rate.

**Fig. 4:**
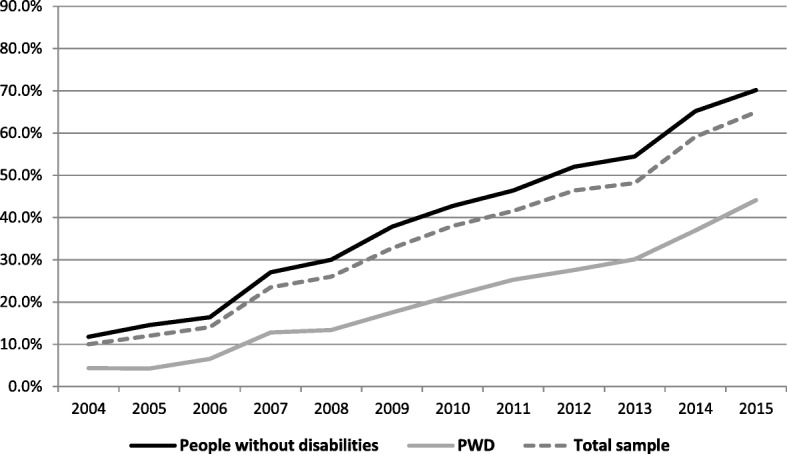
Social media use in the last 3 months between 2003 and 2015

The starting point of social media use was low – only 4% of PWD used social media in 2003, compared to 10% percent among those without disabilities. During the period from 2003 to 2015 the gap between the groups increased: 44% of PWD used social media in 2015 compared to 71% among the respondents without disabilities (Fig. 4). The change in social media use over the 13 years among PWD was much lower (40 percentage points), compared to 61 percentage points in the other group. The odds ratio was more pronounced for people without disabilities (21.7) than for PWD (20.6).

### Multivariate analyses

#### Predicting internet use^1^

In order to compare the two groups for predicting Internet use, separate analyses were performed for respondents with and without disabilities. A logistic regression was performed in three stages. In the first stage the wave of data collection (WDC) and dichotomous PWD variable were entered. In the second stage socio-demographic variables were added. In order to identify changes in the effects of socio-demographic variables over time, in the third stage we added the interactions between WDC and background variables with the most powerful effect on Internet use, according to the research literature: gender, ethnicity (Arabs and immigrants), religiosity, Hebrew language proficiency, education, income and physical and health problems^2^ [19, 26, 29, 51, 54, 57]. The final regression model included only the significant interactional effects in at least one of the groups.

The findings for PWD will be presented first, after which differences between them and sample participants without disabilities will be summarized.

##### The findings for PWD

As can be seen from Table 1, Model 1, the odds of using vs. not using Internet in the preceding 3 months increased over time. The odds of using vs. not using Internet among respondents with physical and health problems that interfere *greatly* were lower compared to those who reported that the problems only interfere. The odds of using vs. not using Internet among Arabs were lower compared to veteran Jews (see Model 2). The odds of using vs. not using Internet in the preceding 3 months were higher among males and employed compared to females and those who were not working. The higher the educational level, Hebrew proficiency and family income, the higher were the odds of using vs. not using Internet. The lower the age, level of religiosity and number of children, the higher were the odds of using vs. not using Internet.

**Table 1:** Logistic regression model: internet use in the last 3 months

	PWD						People without disabilities					
	Model 1		Model 2		Model 3		Model 1		Model 2		Model 3	
	B	Exp(B)	B	Exp(B)	B	Exp(B)	B	Exp(B)	B	Exp(B)	B	Exp(B)
Wave of Data Collection (WDC)	0.17**	1.18	0.23**	1.26	0.29**	1.33	0.18**	1.20	0.24**	1.27	0.36**	1.43
Physical or Health Problem (comparison to “no health problems”)												
The problem doesn’t interfere at all							−0.13**	0.88	0.00	1.00	0.00	1.00
The problem doesn’t interfere very much							−0.54**	0.58	−0.12**	0.89	−0.12**	0.89
Physical or Health Problem (comparison to “the problem interferes”)												
The problem interferes greatly	−0.52**	0.59	−0.29**	0.75	−0.29**	0.75						
Gender (male = 1)			0.30**	1.34	0.32**	1.37			0.28**	1.32	0.48**	1.61
Ethnicity (comparison to veteran Israelis)												
Immigrants			0.11	1.11	−0.45**	0.64			−0.20**	0.82	−0.42**	0.66
Arabs			−0.76**	0.47	−0.93**	0.40			−0.79**	0.46	−1.16**	0.31
Age			−0.06**	0.94	−0.05**	0.95			−0.05**	0.95	−0.04**	0.96
Locality (center = 1)			0.07	1.07	0.07	1.07			0.06*	1.06	0.06*	1.06
Family income			0.08**	1.08	0.09**	1.09			0.08**	1.08	0.07**	1.07
Religiosity			−0.50**	0.61	−0.53**	0.59			−0.64**	0.53	−0.47**	0.63
Education			0.47**	1.61	0.47**	1.60			0.55**	1.74	0.55**	1.74
Number of children			−0.08**	0.93	−0.08**	0.92			−0.11**	0.89	−0.11**	0.90
Marital status (married = 1)			0.02	1.02	0.01	1.01			−0.15**	0.86	−0.17**	0.85
Hebrew proficiency			0.55**	1.74	0.58**	1.78			0.46**	1.59	0.48**	1.62
Employment (employed = 1)			0.40**	1.49	0.39**	1.48			0.27**	1.32	0.27**	1.31
Arabs * WDC					0.02	1.02					0.06**	1.06
Immigrants * WDC					0.09**	1.09					0.05**	1.05
Age * WDC					0.00**	1.00					0.00**	1.00
Gender * WDC					0.00	1.00					−0.04**	0.97
Religiosity *WDC					0.00	1.00					−0.03**	0.97
Family income*WDC					0.00	1.00					0.00**	1.00
Constant	−1.09**	0.34	−1.74**	0.18	−2.17**	0.11	−0.02	0.98	−1.29**	0.27	−2.05**	0.13
Cox & Snell R Square	0.09		0.40		0.40		0.08		0.33		0.34	
Nagelkerke R Square	0.13		0.54		0.54		0.12		0.49		0.49	

* *p* < .05, ** *p* < .01

As can be seen from Model 3, the positive interaction between immigrants and WDC coupled with the negative main effect indicate that the effect of time is stronger among immigrants than among veteran Jews. When considering the amplitude of effects of these variables we can conclude that in 2015, the odds of using vs. not using Internet among immigrants were higher compared to those of veteran Jews, when controlling for socio-demographic variables. The positive interaction between age and WDC coupled with the negative main effect indicates that over time the age-based digital divide diminished, but was not closed. The insignificant effects of the interaction between WDC and Arabs, gender, Hebrew proficiency, family income, religiosity and health problems indicate that the effects of these variables did not change over time.

##### Differences between the groups

The main differences between the groups were found in the interactional effects: significant interactional effects of Arabs, gender, religiosity and family income were found among respondents without disabilities, while among PWD such effects were insignificant. Summarizing interactional effects among people without disabilities we can conclude that the gender gap was closed in 2015; the gap between Arabs and veteran Jews diminished and the gaps based on religiosity and family income increased.

Comparing the quality measures of the models in both groups it should be noted that among the PWD group, socio-demographic variables explain about 54% of variance for the Internet use variable and about 49% among respondents without disabilities.

#### Predicting human and social capital-enhancing forms of internet use

In order to predict the two types of digital uses (human and social capital-enhancing forms of Internet use), logistic and ordinal regressions were conducted in stages. The independent variables were introduced to these models as in the logistic regression described above. These regressions analyzed only Internet users.

##### Predicting human capital-enhancing forms of internet use (seeking information)

*Findings among PWD*. As can be seen from Table 2, Model 1, among Internet users the odds of using Internet in human capital-enhancing ways increased over time. The odds of using Internet in human capital-enhancing ways among respondents with physical and health problems that interfere *greatly* were lower compared to those reported that the problems merely interfere. The odds of seeking and using Internet in human capital-enhancing ways among Arabs were lower compared to veteran Jews (see Model 2), while the differences between immigrants and veteran Israelis were insignificant. The odds of using Internet in human capital-enhancing ways were higher among males and married individuals. The higher the age and number of children, the lower were the odds of using Internet in human capital-enhancing ways. The higher the education and Hebrew proficiency levels, the higher were the odds of using Internet in human capital-enhancing ways. The effects of other socio-demographic variables on the odds of human capital-enhancing uses were insignificant. All interactional effects were insignificant, indicating that the effects of socio-demographic variables on this type of uses were stable over time.

**Table 2:** Logistic regression model: internet use for seeking information in the last 3 months among Internet users

	PWD				People without disabilities			
	Model 1		Model 2		Model 1		Model 2	
	B	Exp(B)	B	Exp(B)	B	Exp(B)	B	Exp(B)
Wave of Data Collection (WDC)	0.05**	1.05	0.07**	1.07	0.04**	1.04	0.06**	1.07
Physical or Health Problem (comparison to “no health problems”)								
The problem doesn’t interfere at all					0.21*	1.23	0.14	1.15
The problem doesn’t interfere very much					0.12	1.13	0.20#	1.22
Physical or Health Problem (comparison to “the problem interferes”)								
The problem interferes greatly	−0.29**	0.75	−0.22#	0.80				
Gender (male = 1)			0.29**	1.34			−0.30**	0.74
Ethnicity (comparison to veteran Israelis)								
Immigrants			0.13	1.14			0.02	1.02
Arabs			−0.67**	0.51			−0.54**	0.58
Age			−0.02**	0.98			−0.01**	0.99
Locality (center = 1)			−0.22*	0.80			0.08	1.08
Family income			0.02	1.02			0.03**	1.03
Religiosity			−0.08	0.92			−0.31**	0.73
Education			0.29**	1.34			0.22**	1.25
Number of children			−0.10*	0.90			− 0.09**	0.92
Marital status (married = 1)			0.38**	1.46			0.13*	1.14
Hebrew proficiency			0.33**	1.39			0.38**	1.47
Employment (employed = 1)			−0.19	0.83			−0.12*	0.88
Constant	2.35**	10.45	0.88	2.41	2.67**	14.43	1.54**	4.65
Cox & Snell R Square	0.00		0.03		0.00		0.03	
Nagelkerke R Square	0.01		0.07		0.00		0.08	

#*p* < 0.1,* *p* < .05, ** *p* < .01

*Differences between the groups.* The effects of family income, religiosity and employment on the odds of using Internet in human capital-enhancing ways were significant among people without disabilities and insignificant in the PWD group. Males in the PWD group were more likely to use Internet in human capital-enhancing ways compared to females from this group, while among people without disabilities the opposite gender pattern effect was found. PWD from the periphery were more likely to use Internet in human capital-enhancing ways, while among those without disabilities the effect of locality was insignificant.

It is important to note that the quality measures of the model in the sample of Internet users were only about 7% and 8% in both groups (compared to 54% and 49% respectively for predicting Internet use). This may be a result of a self-selection process among Internet adopters compared to the general population. In other words, the main selection on the basis of socio-demographic characteristics occurs in the first level digital divide, whereas among Internet users the distinguishing ability of these characteristics is much lower.

##### Predicting social capital-enhancing forms of internet use

*Findings among PWD*. As can be seen from Table 3, Model 1, the odds of using Internet in social capital-enhancing ways increased over time. The odds of using Internet in social capital-enhancing ways among respondents with physical and health problems that interferes *greatly* were lower compared to those who reported that the problems merely interfere, however after controlling for socio-demographic variables this gap became insignificant (see Model 2). Arabs were less likely to use Internet in social capital-enhancing ways compared to veteran Jews, while the gap between immigrants and veteran Jews was insignificant. The lower the age, religiosity and number of children, the higher were the odds of using Internet in social capital-enhancing ways. The higher the education, family income and Hebrew proficiency level, the higher were the odds of using Internet in social capital-enhancing ways. Married respondents and periphery residents were less likely to use Internet in social capital-enhancing ways compared to other marital statuses and center residents. In addition no significant interactional effects were found between wave of data collection and socio-demographic variables, i.e., the effects of the socio-demographic variables were stable over time.

**Table 3:** Ordinal regression model: social media use in the last 3 months among Internet users

		PWD				People without disabilities					
		Model 1		Model2		Model 1		Model2		Model 3	
		Estimate	Std. Error	Estimate	Std. Error	Estimate	Std. Error	Estimate	Std. Error	Estimate	Std. Error
Threshold	[soc_use = .00]	−0.63**	0.06	−1.55**	0.25	−0.92**	0.02	−1.81**	0.11	−0.75**	0.14
	[soc_use = 1.00]	1.34**	0.06	0.73**	0.25	1.54**	0.02	1.00**	0.11	2.04**	0.14
Location	Wave of Data Collection (WDC)	0.15**	0.01	0.21**	0.01	0.20**	0.00	0.26**	0.00	0.41**	0.01
	Physical or Health Problem (comparison to “no health problems”)										
	The problem doesn’t interfere at all					−0.15**	0.04	0.02	0.04	0.03	0.04
	The problem doesn’t interfere very much					−0.05	0.04	0.05	0.04	0.05	0.04
	Physical or Health Problem (comparison to “the problem interferes”)										
	The problem interferes greatly	−0.17**	0.05	−0.07	0.06						
	Gender (male = 1)			−0.01	0.05			0.06**	0.02	0.40**	0.05
	Ethnicity (comparison to veteran Israelis)										
	Immigrants			0.06	0.10			0.22**	0.04	0.23**	0.04
	Arabs			−0.70**	0.11			−0.53**	0.04	−0.55**	0.04
	Age			−0.03**	0.00			−0.03**	0.00	−0.01**	0.00
	Locality (center = 1)			0.15**	0.05			0.11**	0.02	0.11**	0.02
	Family income			0.01**	0.00			0.01**	0.00	0.01**	0.00
	Religiosity			−0.35**	0.04			−0.32**	0.01	−0.12**	0.03
	Education			0.24**	0.02			0.20**	0.01	0.14**	0.02
	Number of children			−0.09**	0.02			−0.11**	0.01	−0.11**	0.01
	Marital status (married = 1)			−0.29**	0.07			−0.44**	0.03	−0.47**	0.03
	Hebrew proficiency			0.09*	0.04			0.05**	0.02	0.06**	0.02
	Employment (employed = 1)			−0.09	0.06			−0.02	0.03	−0.02	0.03
	Age*WDC									0.00**	0.00
	Gender*WDC									−0.05**	0.01
	Religiosity*WDC									−0.03**	0.00
	Education*WDC									0.01**	0.00
	Cox & Snell R Square	0.07		0.20		0.13		0.24		0.24	
	Nagelkerke R Square	0.08		0.23		0.15		0.28		0.28	
	McFadden	0.04		0.11		0.07		0.14		0.15	

* *p* < .05, ** *p* < .01

*Differences between the groups.* In the group without disabilities, males and immigrants were more likely to be social Internet users, compared to females and veteran Jews, whereas among PWD these differences were insignificant. In the group without disabilities the positive interactional effect between age and WDC coupled with the negative main effect indicate that age based digital divide diminishes over time, whereas among PWD differences by age are stable over time. The negative interactional effect between religiosity and WDC coupled with the negative main effect indicate that differences between religiosity groups among people without disabilities increased over time, while among PWD these differences were stable over time. The amplitude of the negative interactional effect between gender and WDC coupled with the positive main effect indicate that over time the gender based gap among the group without disabilities was closed. The positive interactional effect between education and WDC coupled with the positive main effect indicate that the education based digital divide increased over time, whereas among PWD the effect of education did not change.

In studying quality measures of the model it can be seen that the socio-demographic variables explained about 23% of variance for the dependent variable among PWD and about 28% among people without disabilities.

## Discussion

The population of PWD is defined as one of the main risk groups in ICT adoption [22, 23]. According to our findings, in the period from 2003 to 2015 the percentage of Internet access and digital uses increased continuously in the disabled population and the odds ratio among them was similar to the group without disabilities. In terms of the social model of disability this is a promising finding: once disabled persons become Internet users, they have access to a common open space, helping them break down barriers that exist in physical and social environments in the physical non-ITC world [37].

During the period under study the absolute gap between groups was preserved. However, it should also be noted that the gap between the two groups might in reality be higher due to positive self-selection in the PWD group – only those who were mentally and physically capable of participating in the one-hour face-to face CBS surveys were included in the sample.

Our study investigated two types of capital-enhancing digital uses: human capital and social capital. According to the research literature capital-enhancing digital uses offer people greater opportunities and resources for advancing their careers, work, education and social status. However, in our database the majority of PWD don’t work; therefore career advancement and mobility in the labor market are less relevant for them. Preserving cognitive ability, the ability to absorb new information, lifelong learning, social support and exposure to current events are very important for the overall well-being of people excluded from daily interactions and challenges in the workplace [32, 73]. In this context it can be presumed that among PWD who don’t work human capital promotes the additional purpose of maintaining full functioning in life. The term “capital-enhancing digital uses” adjusted for people with disabilities may also include benefiting from eHealth opportunities, utilization of healthcare services, the possibility of near instantaneous communication across the globe which provides a source of health information, forums for health issue discussions and a vehicle for new social relations with people experiencing the same problems or with support groups. Therefore the concept of human capital can be broadened to include issues pertaining to the preservation of health and quality of life for this group.

As for the human capital-enhancing uses as defined in the literature (seeking information) it should be noted that almost all of the Internet users from both groups surfed the Internet to seek information. Moreover, our descriptive findings among Internet users show that the advantage of the group without disabilities was low and stable over time. The percentage of e-mail and social media use among Internet users was lower (compared to information seeking) and stable over time with moderate differences between the groups.

The findings of the multivariate analysis show that the first-level digital divide between the groups, after controlling for socio-demographic variables, increased over time. We found that socio-demographic variables were much more powerful in predicting Internet use vs non-use among the total population of both groups, compared to predicting capital-enhancing digital uses among Internet users. This pattern was more pronounced in the PWD group, compared to the group without disabilities. A prominent first level digital divide coupled with a moderate second level gap, when added to physical impairments and limitations that technically hinder computer and Internet use, may be explained by the compensation model of disability. For people who have limited exposure to their social environment or who experience dissatisfaction with their face-to-face social interactions, gaining access to Internet (and the ability to use it) is tantamount to opening a new world: the Internet becomes their “real” life and they try to utilize its potential to the maximum.

As for trends in the effects of socio-demographic variables over time in the PWD group, we found most of the effects to be stable, thus the gap between Jews and Arabs as well as differences on the basis of gender, religiosity and income are expected to be maintained in the future. This is in contrast to the group without disabilities, where the effects of socio-demographic variables on Internet access have changed over time. The promising news in these findings is that in contrast to the group without disabilities in which religiosity and income gaps increased over time, among PWD the situation did not worsen.

Our findings indicated that belonging to certain groups within the PWD group intersected with additional risk factors. One such factor was affiliation to ethnic and national minorities. In contrast to veteran Jews, Arabs experienced the additional disadvantage of belonging to a minority group, which can be a source of e-exclusion. Our findings indicated both first and second level digital divides between veteran Jews and Arabs with disabilities. In contrast to the group without disabilities in which the disadvantage of Arabs diminished over time, among Arabs with disabilities the gap was stable during the 13 years. As for immigrants, the pace of Internet adoption among them was faster, compared to veteran Jews, and over time initial between-group differences in Internet use were eliminated. However, the advantage of immigrants over veteran Jews in social capital-enhancing digital uses which was found among the group without disabilities was not identified in the PWD group.

Another important factor was gender: women were less likely to access and use Internet in human capital-enhancing ways compared to men. This disadvantage remained stable among PWD after controlling for socio-demographic factors. In this group the gender gap in Internet access and human capital-enhancing digital uses is likely to remain over time due to the similar pace of adoption by both genders. This stands in contrast to the group without disabilities in which the disadvantage of females in Internet access was eliminated over time because of their faster rate of adoption of technology.

Yet another risk factor was SES: respondents with higher education and family income were more likely to adopt the Internet, compared to those with lower SES. The effects of income and education among PWD were stable over the time, i.e., the disability based digital divide continued to intersect with socio-economic disparities.

A relatively strong risk factor was religiosity. In religious and very religious (ultra-orthodox in the Jewish sector) communities, Internet adoption and use were significantly lower compared to traditional and secular populations. It is possible to discern an attitude of suspicion towards all aspects of communication technologies in religious and ultra-religious circles. The ultraorthodox prefer not to use the internet and not be exposed to content they see as harmful [61]. However, the findings among PWD were more optimistic, compared to those among people without disabilities: the effect of religiosity on human capital-enhancing digital use was insignificant. In other words, after Internet adoption religious people with disabilities may gain as much exposure to health and other important information as their secular peers.

An additional disadvantaged group is that of senior citizens. Although the Internet is particularly important for seniors because it serves as a platform for interpersonal communication, maintaining family bonds (especially across vast distances), and expanding social networks, this group realized its potential less than others. However, significant interactional effects between WDC and age on Internet use indicate that the age based digital divide is diminishing in both groups.

## Conclusions

The current research focused on trends of Internet adoption and digital uses among PWD during the period from 2003 to 2015 in order to identify the socio-demographic characteristics predicting Internet access and digital uses. This was the first study to examine whether the effects of these factors changed over time among PWD.

The paper demonstrates the multi-dimensionality of the digital divide phenomena. From one aspect, access gaps between PWD and those without disabilities are stable. In contrast, e-mail and social media usage gaps still seem to be widening and while the group without disabilities is in its late majority phase, the group with disabilities is only reaching the later phases of early adoption. Our important conclusion is that the more prominent differences between the groups were found in the first level of the digital divide (Internet use vs non-use), whereas between-group differences in the second-level digital divide among Internet users were moderate. Our findings make it possible to identify disadvantaged groups in which the disabilities group intersects with additional risk factors: Arabs, religious people, elderly, respondents from low socio-economic backgrounds. The effects of most of these variables did not change in the period under study.

### Study limitations and recommendations for further research

Study limitations derive from the limitations of the CBS social survey database. In this survey capital-enhancing digital uses were examined without details (purpose of surfing, kind of sites visited, types of social media used by respondents, languages of surfing etc.). Moreover, the human capital-enhancing digital users were measured only by one item. Such limitations do not enable us to examine whether and how research participants use Internet for health issues which are so crucial for further understanding of the eHealth literacy concept among populations with PWD. Further research should address this point.

Our findings show that the severity of physical and health problems had a negative effect on Internet adoption and use. Unfortunately, our database does not allow us to distinguish between different types of disabilities in order to propose more specific practical implications for different types of impairments. This may be an important issue for future research.

## Practical implications

The findings of this study have important implications for researchers, educators, practitioners and policy makers who attempt to promote Internet use among Israeli PWD. Moreover, our findings regarding the effects of age, education, ethnicity and income on Internet adoption and use are relevant not only to the Israeli case but may also be generalized to other countries. Optimal engagement of PWD in fully beneficial Internet use requires adjustments at two levels: ensuring physical availability to computers and full accessibility to PWD Internet sites, and acquiring mastery of digital skills and e-health literacy by PWD.

The Israeli Site Accessibility Law came into force on October 26, 2017, with the goal of making the Internet accessible to the general public, and removing the limitations that existed for PWD. This legislation relates to both public and private service providers. However, when it comes to wider Internet accessibility, many areas remain in which people with disabilities lack effective access to operating systems, software, interfaces, hardware, platforms, and content, for example in the sites of relatively small Israeli businesses, e-books or mobile web and non-web mobile apps [33]. The Ministry of Health should continue to ensure that this law is not circumvented and expand it regarding the above mentioned platforms and content.

As for improving digital and e-Health literacy, we recommend that local authorities find a way to promote courses that focus on promoting digital literacy in general and eHealth literacy in particular in small groups of people of similar age, digital skill level and motor / health problems. It should also be taken into account that among PWD this learning process might be accompanied by emotional or cognitive factors that affect the learning and training process. Caregivers and social workers who are involved in the learning process should be aware of such psychological barriers and provide constant support and encouragement. Training can be given in community or therapeutic centers as part of the treatment a person receives for health problems.

Due to the prolonged interaction of PWD with the professionals who care for them, this latter group may help in exposing their care recipients to the Internet. For example, medical secretaries may show patients how to schedule an appointment with a doctor on the Internet, ask for tests or prepare regular prescriptions. Doctors / nurses can refer their patients to websites that contain databases related to their problems. The inspectors from the Ministry of Health who regularly visit medical institutions can encourage those institutions that have not yet adopted these practices to do so. The relevant databases should be in the patients’ language (Hebrew, Arabic, Russian etc.) using simple non-professional language that can provide patients with support, knowledge and assistance with their illness.

We found that low Internet use is more prevalent among those populations with disabilities that would benefit greatly from eHealth, namely low-income, less educated, Arab minority, and older populations. One of the main disadvantaged groups in terms of Internet access was the disabled Arab population. The results of the current investigation suggest that overcoming digital divides is a complex challenge that goes beyond improving Internet access. Closing digital gaps requires changes in basic social, economic and cultural aspects of the Arab sector on the individual level, i.e., personal motivation, as well as on the community level, including collective socio-cultural preferences. However, in spite of the fact that until recently Arabic was the second official language,^3^ most of the sites belonging to major bodies in the public sector, including important eHealth sites, had little or no information in Arabic and among those that did, the gap between the Hebrew and Arabic versions in terms of scope, currency and quality of language was usually great [1]. Considering the high representation of Arabs among PWD, it is recommended that efforts continue to increase the scope and quality of Arabic language content published on Israeli eHealth sites. The Ministry of Health should supervise implementation of these processes.

In terms of accessibility, this study found a positive correlation between family income and Internet adoption both among respondents with and without disabilities. Despite the continual decline in the cost of digital equipment, it is likely that the expenditures involved in purchasing a computer or Internet access will continue to entail economic constraints. One way to alleviate this problem may be to provide publicly accessible free information technologies, for example, in community clubs, senior citizen clubs, and independent- and assisted- living projects for the disabled. Providing such solutions should be the responsibility of local authorities.

If citizens with disabilities are able to effectively use the diverse opportunities offered by the Internet in general and especially by eHealth services, in the long run they will be more able to maintain their quality of life and prevent risks of disease, deterioration of health and unhealthy behaviors [8, 86]. Moreover, psychologically they may enjoy the fact that they are no longer socially isolated, but are “e-included” and continue to be important and influential members of society.

## Appendix 1

**Table 4:** Socio-demographic characteristics of the sample

		PWD	Without disabilities	Total sample
Ethnicity	Immigrants	22.0%	16.5%	17.7%
	Arabs	17.1%	14.8%	15.3%
	Veteran Jews	60.9%	68.8%	66.9%
Gender	Female	56.5%	50.1%	51.6%
	Male	43.5%	49.9%	48.4%
Locality	Periphery	43.0%	41.2%	41.6%
	Center	57.0%	58.8%	58.4%
Religiosity	Secular	34.5%	42.2%	40.4%
	Traditional	41.0%	33.8%	35.5%
	Religious	18.8%	15.8%	16.5%
	Orthodox	5.8%	8.2%	7.7%
Marital status	Married	62.8%	65.3%	64.7%
	Separated	1.1%	0.7%	0.8%
	Divorced	9.3%	6.1%	6.9%
	Widowed	15.8%	3.3%	6.2%
	Single	11.1%	24.5%	21.4%
Employment status	Doesn’t employed	63.7%	28.3%	36.6%
	Employed	36.3%	71.7%	63.4%
Number of children	No children	12.9%	27.2%	23.8%
	1	11.3%	11.1%	11.1%
	2	23.2%	21.1%	21.6%
	3	19.2%	19.7%	19.6%
	4+	32.1%	18.6%	21.8%
Education	Secondary school completion	31.3%	22.0%	23.8%
	Matriculation certificate	17.7%	23.9%	22.7%
	Post-secondary, non-academic	25.6%	20.8%	21.7%
	BA	12.3%	20.6%	19.0%
	MA	12.0%	11.4%	11.5%
	PhD	1.2%	1.4%	1.3%
Age	Mean	57.95	42.23	45.91
Family income	Mean, NIS	7413.56	11,489.13	10,508.15
Hebrew proficiency	Mean (scale 1–5)	3.66	4.45	4.27

## Appendix 2

**Table 5:** Internet uses among internet users over time

	Seeking information		E-mail		Social media		Social capital forms of internet use, scale 0–2				
	Without disabilities	PWD	Without disabilities	PWD	Without disabilities	PWD	Without disabilities		PWD		
	%	%	%	%	%	%	M	SD	M	SD	t
2003	92.4%	87.8%	79.0%	70.4%	21.9%	21.1%	1.01	0.62	0.91	0.66	2.44*
2004	94.9%	91.6%	79.8%	70.8%	22.9%	23.5%	1.03	0.62	0.94	0.69	2.33*
2005	94.9%	91.9%	81.7%	73.5%	25.9%	19.2%	1.08	0.62	0.93	0.64	4.56**
2006	95.2%	93.0%	82.9%	72.6%	27.5%	26.3%	1.10	0.63	0.99	0.69	3.54**
2007	92.3%	89.6%	84.8%	76.1%	40.6%	40.7%	1.25	0.67	1.17	0.72	2.88**
2008	93.9%	92.6%	89.0%	80.4%	44.2%	43.9%	1.33	0.63	1.24	0.71	2.97**
2009	95.3%	93.3%	89.5%	80.2%	52.2%	48.8%	1.42	0.64	1.29	0.72	4.61**
2010	95.1%	93.7%	87.8%	77.6%	56.6%	52.9%	1.44	0.66	1.3	0.73	5.07**
2011	94.9%	92.9%	89.2%	77.8%	59.9%	56.9%	1.49	0.63	1.35	0.74	5.46**
2012	95.4%	93.3%	87.9%	78.3%	66.7%	59.9%	1.55	0.63	1.38	0.73	6.41**
2013	94.8%	93.2%	90.2%	79.8%	70.1%	64.0%	1.60	0.60	1.44	0.69	7.37**
2014	95.3%	91.4%	87.1%	74.8%	80.2%	72.1%	1.67	0.57	1.47	0.68	8.89**
2015	94.2%	91.1%	85.9%	73.2%	84.8%	79.7%	1.71	0.53	1.53	0.64	8.49**

* *p* < .05, ** *p* < .01

## Footnotes

1 In order to examine differences between people with and without disabilities three regression models were constructed: (1) a logistic regression model predicting internet use among total sample; (2) a logistic regression model predicting information seeking among internet users; (3) an ordinal regression model predicting social media use among internet users. All three regressions were conducted in three stages: in the first stage wave of data collection (WDC) and the dichotomous PWD variable were entered; in the second stage all socio-demographic variables were entered; in the third stage the interactional effect between the WDC and dichotomous PWD variable were included. In all three regressions an initial disadvantage was found for PWD. After controlling for socio-demographic variables this disadvantage remained significant in regressions (1) and (3) and was eliminated in regression (2). In regression (1) for predicting internet use, a negative significant interactional effect coupled with a negative main effect indicates that over time the disadvantage of PWD worsened. The amplitude of the main and interactional effect found in regression (3) indicates that the gap in social media uses in favor of the people without disabilities was preserved over time. In the interest of space, we have omitted these regressions. These results are available from the authors on request.

2 For similar methodology in repeated cross-sectional as well as panel studies see [3, 24, 53, 56, 57, 72].

3 On 19 July 2018, the Knesset passed a basic law under the title Israel as the Nation-State of the Jewish People, which defines Hebrew as “the State’s language” and Arabic as a language with “a special status” in the State.
